# Health App Use Among US Mobile Phone Owners: A National Survey

**DOI:** 10.2196/mhealth.4924

**Published:** 2015-11-04

**Authors:** Paul Krebs, Dustin T Duncan

**Affiliations:** ^1^ New York University School of Medicine Department of Population Health New York, NY United States; ^2^ The Department of Veterans Affairs (VA) New York Harbor Healthcare System New York, NY United States; ^3^ College of Global Public Health New York University New York, NY United States; ^4^ Population Center New York University New York, NY United States; ^5^ Center for Data Science New York University New York, NY United States

**Keywords:** cell phones, mobile apps, telemedicine

## Abstract

**Background:**

Mobile phone health apps may now seem to be ubiquitous, yet much remains unknown with regard to their usage. Information is limited with regard to important metrics, including the percentage of the population that uses health apps, reasons for adoption/nonadoption, and reasons for noncontinuance of use.

**Objective:**

The purpose of this study was to examine health app use among mobile phone owners in the United States.

**Methods:**

We conducted a cross-sectional survey of 1604 mobile phone users throughout the United States. The 36-item survey assessed sociodemographic characteristics, history of and reasons for health app use/nonuse, perceived effectiveness of health apps, reasons for stopping use, and general health status.

**Results:**

A little over half (934/1604, 58.23%) of mobile phone users had downloaded a health-related mobile app. Fitness and nutrition were the most common categories of health apps used, with most respondents using them at least daily. Common reasons for not having downloaded apps were lack of interest, cost, and concern about apps collecting their data. Individuals more likely to use health apps tended to be younger, have higher incomes, be more educated, be Latino/Hispanic, and have a body mass index (BMI) in the obese range (all *P*<.05). Cost was a significant concern among respondents, with a large proportion indicating that they would not pay anything for a health app. Interestingly, among those who had downloaded health apps, trust in their accuracy and data safety was quite high, and most felt that the apps had improved their health. About half of the respondents (427/934, 45.7%) had stopped using some health apps, primarily due to high data entry burden, loss of interest, and hidden costs.

**Conclusions:**

These findings suggest that while many individuals use health apps, a substantial proportion of the population does not, and that even among those who use health apps, many stop using them. These data suggest that app developers need to better address consumer concerns, such as cost and high data entry burden, and that clinical trials are necessary to test the efficacy of health apps to broaden their appeal and adoption.

## Introduction

As of 2015, 64% of the overall US population and 82% of persons aged 18-49 years owned an app-enabled mobile phone [1]. Additionally, 15% of the population now owns a mobile phone-connected wearable device, such as a Fitbit or smartwatch [2]. As such, it is not surprising that mobile phone apps, which focus on health, fitness, or medical care (ie, health apps), have become highly popularized. Over 40,000 health-related apps were available for download from the Apple iTunes store alone as of 2013 [3]. While many people may first associate health apps with fitness and diet-focused apps (eg, Lose It!, MapMyFitness, and MyFitnessPal), the category spans numerous domains inclusive of prevention/lifestyle, self-diagnosis, provider directories, diagnosis/education, prescription filling, and treatment compliance. For instance, health apps such as iStayHealthy and PozTracker exist to track HIV medication and personal health statistics (eg, CD4 count), and WellDoc exists for diabetes management.

The field of mobile health apps is still in a nascent stage and is characterized by a number of limitations, both in terms of sophistication of the apps themselves as well as in knowledge of consumer profiles. Most health apps have not been designed with input from health care and behavior change professionals. A detailed analysis found that among apps classified as "health and fitness" or "medical," only one-fifth offered the possibility of facilitating actual behavioral or physical changes versus nonevidence-based gimmicks or simple information provision [3]. Most apps advertised as health related have limited functionality, “do little more than provide information,” and do not offer tracking or data input options [3]. Indeed, a recent review of physical activity apps found that none provided evidence-based guidelines for aerobic activity and only 2% did so for resistance training [4].

Data is also limited with regard to consumer opinions and usage patterns of health-related apps. One survey of pediatric practices in New York found that 35% of parents and 17% of teens had used a health-related app [5], but this study primarily focused on adolescents and recruited a small sample (n=148). Others have only examined specific apps, for instance, reporting demographics of MapMyFitness users [6], or have surveyed specific populations such as those with health insurance [7].

Additionally, developers typically do not release information on the number of users and the extent to which consumers continue to use apps over time. Download statistics and feedback ratings from app stores are available, but such data lack validity and do not provide detailed information with regard to important information such as demographics of users, primary reasons people download health apps, barriers to use, consistency of use, and reasons for noncontinuance. Given this lack of population-based data on health apps, the goal of this study was to survey multiple aspects of health app use from a consumer perspective using a diverse sample of mobile phone users in the United States.

## Methods

### Study Design and Sample

A cross-sectional survey of 1604 mobile phone users in the United States was conducted in June 2015. Toluna, a survey management company, hosted the survey and recruited participants. Toluna identified potential participants by emailing their existing panel of respondents and by posting targeted online advertisements. Once respondents clicked on the Toluna survey link, they were screened for the following eligibility criteria: aged 18 years or older, spoke English, and owned a mobile phone. Those who were eligible completed an online informed consent document. As surveys of technology use tend to include more females and persons from higher socioeconomic backgrounds, we employed Toluna’s quota sampling capability to attempt to achieve the following demographic distribution: 50% female; 50% having completed high school or fewer years of education; 60% earning less than US $50,000 per year; and 30% Latino/Hispanic, 30% black, 30% white, and 10% Asian or other. Respondents received points for completing the survey through Toluna, which they could redeem for rewards. Respondents were required to indicate an answer before moving on to the next question, but could review and change previous answers. Each question appeared individually on a unique page. Start and stop times were assessed and only surveys completed within less than 120 minutes were analyzed. Toluna sent the authors a deidentified data file. The New York University School of Medicine Institutional Review Board approved all study procedures.

### Survey Items

The survey consisted of 36 questions encompassing the following domains: (1) sociodemographic characteristics, (2) history and reasons for health app use/nonuse, (3) perceived effectiveness of health apps, (4) reasons for stopping use, and (5) standard health questions (eg, tobacco use, weight, height, medical diagnoses, physical activity, and eating behaviors). As there was no precedent for app use items, our research team developed app-related questions and field-tested them among a diverse team of colleagues with expertise in survey development. Questions were presented to each participant in the same order as there was a necessary logical order; however, the order of within-item responses was randomly assigned to reduce response-set bias. The survey took participants an average of 9 minutes to complete.

### Data Analysis

Descriptive statistics were calculated for all items. Open-ended responses were examined qualitatively using thematic analysis. In particular, we used an inductive qualitative analytical approach [8]. Two coders examined the data and determined overarching categories. Representative quotes were selected for each theme. Regarding the quantitative analysis, given the high prevalence (>50%) of app users (primary outcome), we employed multivariable Poisson regression—GENMOD procedure in SAS (SAS Institute Inc)—using methods outlined by Zhao [9]. The analysis assessed the following demographic correlates of ever having downloaded a health app: age, sex, race/ethnicity, education, income, body mass index (BMI)—underweight, overweight, or obese versus normal weight—and self-reported diagnosis of any current chronic health condition. Statistical significance was determined by *P* values less than .05. All statistical analyses were conducted using SAS version 9.3 (SAS Institute Inc).

## Results

### Demographic and Health Characteristics

**Figure 1:**
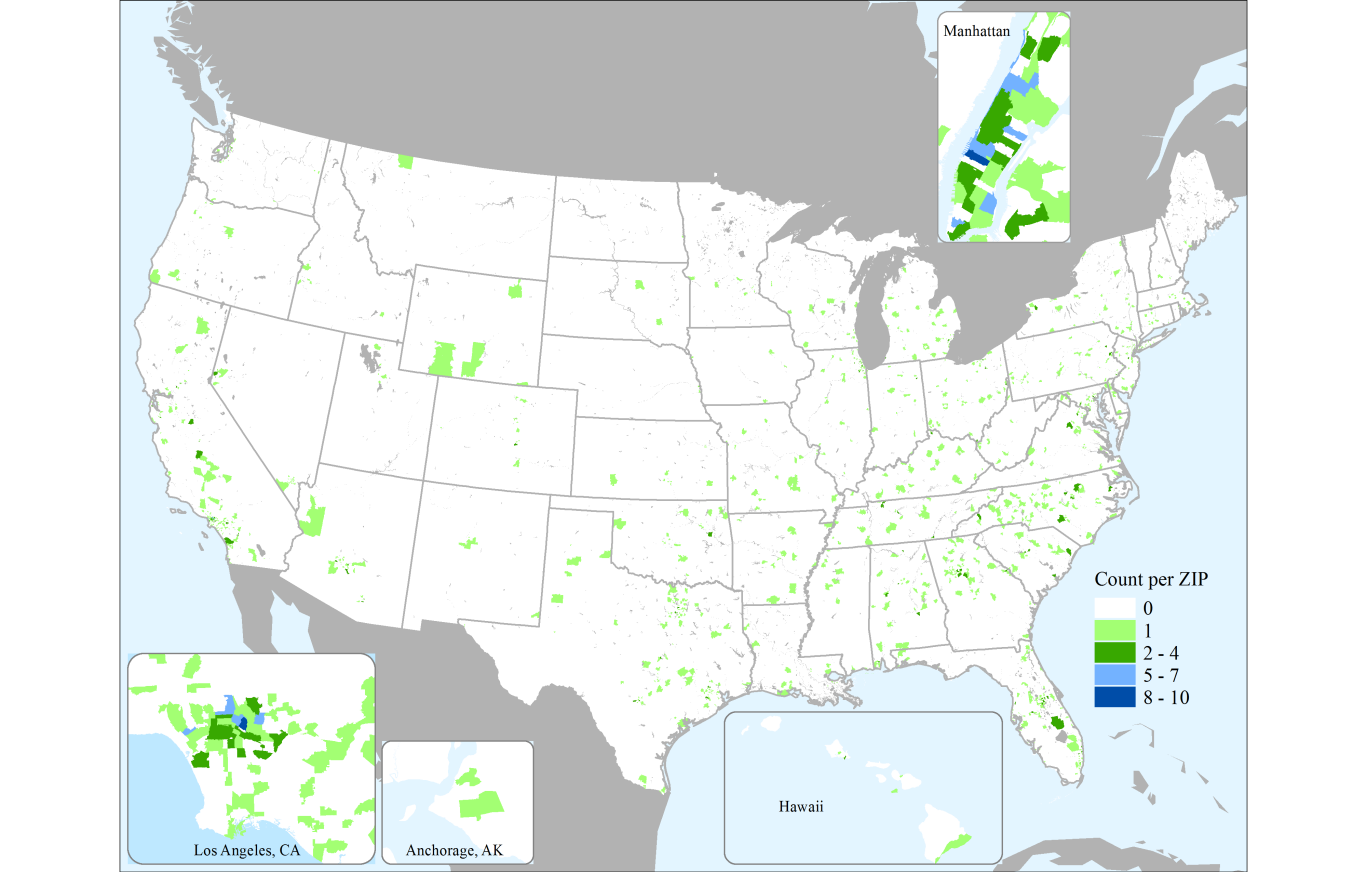
US distribution of sample by ZIP code.

A total of 7189 people visited the survey page, and 6871 (95.58%) agreed to participate. Of the 6871 who agreed to participate, 2089 (30.40%) completed the entire survey and 485 of these (23.22%) were removed due to overfilling of the demographic quotas. The mean age of the analytic sample was 40.1 years (SD 15.7) and ranged from 18-81 years. A total of 49.56% (795/1604) of the sample was female, and 60.38% (960/1590) had annual incomes of less than US $50,000 per year. In terms of race/ethnicity, the sample was 25.44% black (408/1604), 7.11% Asian (114/1604), 35.47% white (569/1604), 27.87% Latino/Hispanic (447/1604), and 2.87% other (46/1604) (Table 1). Figure 1 shows the spatial distribution of the sample throughout the United States by self-reported residential ZIP codes.

In terms of health, only 16.40% (263/1604) reported they never engaged in physical activity for at least 15 minutes, 34.04% (546/1604) had BMIs in the normal range, and 62.03% (995/1604) were overweight or obese. Only 50.69% (813/1604) considered themselves overweight, and 51.12% (820/1604) thought their health was very good or excellent. Almost a quarter (353/1604, 22.01%) smoked cigarettes every day and 12.16% (195/1604) on at least some days. The most prevalent medical diagnoses respondents reported having were hypertension (364/1604, 22.69%), high cholesterol (319/1604, 19.89%), depression (267/1604, 16.65%), obesity (198/1604, 12.34%), and diabetes (163/1604, 10.16%) (Table 1; see Multimedia Appendix 1 for the full list of items and responses).

**Table 1:** Sociodemographic and health characteristics of the sample (abridged ^a^) (n=1604).

Item	Characteristic	n (%)
Sex	Female	795 (49.56)
Race/ethnicity		
	African American or black	408 (25.44)
	Asian American or Asian	114 (7.11)
	White or Caucasian	569 (35.47)
	Native American/Pacific islander	20 (1.25)
	Latino/Hispanic	447 (27.87)
	Other	46 (2.87)
Born in the United States	Yes	1445 (90.09)
Education		
	Less than 12th grade	79 (4.93)
	High school degree or GED^b^	722 (45.01)
	Some college/vocational school/apprenticeship	399 (24.88)
	Bachelor’s degree	276 (17.21)
	Graduate degree (master's, PhD, MD, etc)	128 (7.98)
Household income		
	Less than US $25,000	463 (28.87)
	US $25,000-49,999	497 (30.99)
	US $50,000-74,999	218 (13.59)
	US $75,000-99,999	195 (12.16)
	US $100,000+	231 (14.40)
Region of country (n=1594) ^c^		
	Northeast	322 (20.20)
	Midwest	242 (15.18)
	South	636 (39.90)
	West	394 (24.72)
In general, would you say your health is...?		
	Poor	44 (2.74)
	Fair	204 (12.72)
	Average	536 (33.42)
	Very good	603 (37.59)
	Excellent	217(13.53)
Body mass index (kg/m^2^)		
	<18.5 (underweight)	63 (3.93)
	18.5-24.9 (normal)	546 (34.04)
	25-29.9 (overweight)	438 (27.31)
	≥30 (obese)	557 (34.73)
Do you consider yourself to be...?		
	About the right weight	683 (42.58)
	Underweight	108 (6.73)
	Overweight	813 (50.69)

^a^ Table 1 is an abridged version; see Multimedia Appendix 1 for the full list of items and responses.

^b^GED: General Educational Development.

^c^Not all participants provided ZIP code information.

### Health App Usage

Most respondents used mobile phones sold by Apple (565/1604, 35.22%) or Samsung (567/1604, 35.35%). AT&T was the most popular service provider (420/1604, 26.18%) followed by Verizon (342/1604, 21.32%) and T-Mobile (311/1604, 19.39%). With regard to health app use, 58.23% (934/1604) had downloaded an app to track their health in the past, with 41.6% (389/934) having downloaded more than five health-related apps. The most frequent reasons people reported for downloading health apps were to track how much physical activity they were getting (493/934, 52.8%), to track what they ate (445/934, 47.6%), to lose weight (437/934, 46.8%), and to learn exercises (318/934, 34.0%).

The majority (612/934, 65.5%) of respondents opened their health apps at least once per day, and 44.4% (415/934) used their apps for 1-10 minutes. Belief that apps keep data secure was high, with 44.0% (411/934) and 34.2% (319/934) reporting they moderately or very much trusted their apps’ data security. Similarly, 44.5% (416/934) and 36.8% (344/934) thought that apps recorded data with moderate or high accuracy, respectively. The three most popular methods by which respondents learned about apps was searching the app store (327/934, 35.0%), from friends/family (287/934, 30.7%), and Web searches (170/934, 18.2%). Only 20.37% (210/1031) of respondents reported that a doctor had recommended a health app to them. A large proportion noted that they would never pay anything for a health app (662/1604, 41.27%), 20.26% (325/1604) would pay up to US $1.99, and 22.76% (365/1604) would pay at most between US $2.00 and US $5.99.

### Nonuse and Reasons for Discontinuing Use

Among the 41.77% (670/1604) who had never downloaded a health app, the most important reasons they had not done so were lack of interest (181/670, 27.0%), high cost (156/670, 23.3%), lack of trust in apps collecting their data (103/670, 15.4%), concern that they would use too much data (85/670, 12.7%), and belief that they did not need a health app (73/670, 10.9%). A large portion of the sample (427/934, 45.7%) reported that they downloaded health apps they no longer use. The most frequent reasons for discontinuance were the following: took too much time to enter data (190/427, 44.5%), loss of interest (173/427, 40.5%), hidden costs (154/427, 36.1%), apps were confusing to use (140/427, 32.8%), and did not like that the apps shared their data with friends (124/427, 29.0%) (Table 2; see Multimedia Appendix 2 for the full list of items and responses).

### Preferences for App Features

#### Overview

In terms of potential features and uses for health apps, 57.36% (920/1604) would be somewhat or very interested in the ability to make appointments with, or write to, their doctors, and 62.16% (997/1604) would like to view their medical records. Less than 10% of respondents used these features at the time of the study. Three primary themes emerged from the analysis of open-ended items, as discussed below.

#### Weight Loss, Calorie Tracking, Nutrition, and Physical Activity

The majority of comments concerned the intersection of food intake, physical activity, and weight management. A major theme was that participants wanted apps to provide more specific and personalized recommendations, regarding exercises/activities and what to eat than are currently available. For instance, a number of respondents noted they wanted an app to assess their health history, and for the app to tell them what exercises they should do and what they should and should not eat. For instance, participants wanted an app to tell them the following: “Remind me what food I have to eat every single day,” “Tell me when I am eating the wrong food,” and “Suggest exercises, customize workouts to fit my goals and needs.” Generally, they wanted apps that helped them reach specific exercise and nutrition goals rather than just "lose weight." Tracking was also an important theme, with participants wanting more accurate and easier-to-use methods of showing how many calories they consume and burn daily. Participants stated, “I would want it to tell me how many calories I need to consume for the day and keep track so I know when I need to stop eating,” “I wish I could take a picture of my food and it would count the calories for me,” and “[I would want the app to] Speak to me if I tell it what I ate and tell me how many calories I ate then tell me how many calories I have left to eat that day.” A number of participants also wanted the apps to help keep them motivated, particularly using humor and encouragement. Specifically, they noted, “I would like it to have something entertaining for me to focus on should I ever get unwanted food cravings as a distraction,” and they wanted it to have “...an alarm to remind me to get moving, 'it's time for your 30 min. walk, hop to it chubby'... it has to be funny !!!”

**Table 2:** Characteristics of health app use (abridged ^a^).

Survey item	Response category	n (%)
3. Have you ever downloaded an “app” to track anything related to your health? (n=1604)	Yes	934 (58.23)
4. How many health-related smartphone apps have you used?^a^ (n=934)		
	1-5 apps	545 (58.4)
	6-10 apps	104 (11.1)
	11-20 apps	160 (17.1)
	More than 20	125 (13.4)
5. Please check off all the reasons you have used health apps. (check all that apply) ^a^ (n=934)		
	Track how much activity/exercise I get	493 (52.8)
	Help me watch what I eat	445 (47.6)
	Weight loss	437 (46.8)
	Show/teach me exercises	318 (34.0)
	Track a health measure	266 (28.5)
6. Rank the most important reasons you have not downloaded a health app. ^a^ (n=670)		
	I’m just not interested in health apps	181 (27.0)
	They cost too much to buy	156 (23.3)
	I don’t trust letting apps collect my data	103 (15.4)
	My health is fine and I don’t need one	73 (10.9)
	They would use too much of my data	85 (12.7)
	They are too complicated to use	72 (10.7)
7. What would be the maximum amount you would pay for a health-related app? ^a^ (n=1604)		
	I wouldn't pay anything	662 (41.27)
	US $1-US $3.99	400 (24.94)
10. How much do you trust that your health apps automatically record your data accurately? ^a^ (n=934)		
	Moderately trust	416 (44.5)
	Very much trust	344 (36.8)
13. To what extent do you think health apps have improved your health? (n=934)		
	Made worse/didn't help at all	98 (10.5)
	Just a little bit/somewhat improved	563 (60.3)
	Very much improved	273 (29.2)
14. Which health apps do you currently have on your phone? (free text) ^a^ (n=934)		
	Walgreens	123 (13.2)
	Fitbit	107 (11.5)
	Weight Watchers	59 (6.3)
	Web MD	36 (3.9)
	Nike+	34 (3.6)
15. Are there any health apps you downloaded and no longer use? (n=934)	Yes	427 (45.7)
16. What reasons do you no longer use them? (check all that apply) ^a^ (n=427)		
	Takes too much time to enter data	190 (44.5)
	Lost interest	173 (40.5)
	There were hidden costs	154 (36.1)
20. Has a doctor ever recommended you use a health app? (n=1031)	Yes	210 (20.37)

^a^ Table 2 is an abridged version; see Multimedia Appendix 2 for the full list of items and responses.

#### Communication With Medical Care Systems

The next most common theme involved improved communication with the health care system. Many participants simply wanted an app to show their medical records, while others wanted to easily make appointments and to engage in two-way communication with their doctors. Having reminders for medication taking and appointments was also commonly mentioned. Some respondents wanted an all-in-one system such that they did not have to use multiple medical-related apps. Participants described a desire for an app that would keep track of all their vital statistics (weight, diet, sleep, etc) to better communicate with their doctors during appointments (ie, linking with and inputting these data into their medical record). For instance, one participant stated, “I would want to be able to easily schedule appointments with my doctor, write to my doctor with concerns, have my doctor be able to respond to my concerns, view my medical records, be able to track my medications.” Another participant noted that interaction with the electronic health record (EHR) would enable “...graphs showing my health as time passes.”

#### Medical Monitoring

A number of participants mentioned they wanted an app to track symptoms and make possible diagnostic suggestions. They wanted to “...have full access to my health records and the doctors I have to see, to diagnose some health concerns or issues by symptoms,” and to “Help me to diagnose myself by typing in my symptoms” or “...jotting down symptoms that are ailing me at the time, so I could send them to my doctor.”

### Correlates of Having Downloaded an App

Having downloaded a health app was significantly (*P*≤.05) related to younger age, being Latino/Hispanic, having a higher income, having greater than high school education, and obesity. There was also a trend for African Americans to be more likely to use health apps (relative risk [RR]=1.12, *P*=.07). No significant relationship was found with sex or history of chronic disease after controlling for other variables (Table 3).

**Table 3:** Multivariable correlates of health app usage.

Variable		RR^a^ (95% CI)	*P*
Intercept			<.001
Female (vs male)		0.98 (0.90-1.07)	.64
Age in years (each year)		0.98 (0.97-0.98)	<.001
Race/ethnicity (vs white)			
	African American or black	1.12 (0.99-1.26)	.07
	Asian American or Asian	0.94 (0.78-1.13)	.51
	Latino/Hispanic	1.19 (1.06-1.33)	.002
Education (vs less than high school)		1.12 (1.03-1.22)	.01
Income (vs US $50,000-$74,999)			
	US $100,000+	1.33 (1.16-1.51)	<.001
	US $75,000-$99,999	1.32 (1.16-1.50)	<.001
	US $25,000-$49,999	0.98 (0.85-1.13)	.81
	Less than US $25,000	0.79 (0.67-0.93)	.004
Diagnosed with chronic disease		0.97 (0.89-1.05)	.42
BMI ^b^ (vs normal weight)			
	Underweight	0.88 (0.75-1.05)	.15
	Overweight	1.09 (0.98-1.21)	.10
	Obese	1.11 (1.01-1.22)	.02

^a^RR: relative risk.

^b^BMI: body mass index.

## Discussion

### Principal Findings

This study examined health app usage among a socioeconomically and geographically diverse sample of US mobile phone users. A little over half (934/1604, 58.23%) of mobile phone users had downloaded a health-related mobile app. Fitness and nutrition were the most common categories of health apps used, with most respondents using them at least daily. A fairly large proportion of respondents, however, had not used health apps. Common reasons for not doing so were lack of interest, cost, and concern about apps collecting their data. Persons more likely to use health apps tended to be younger, tended to have higher income and greater education, were Latino/Hispanic, and had a BMI in the obese range. Latinos/Hispanics were 20% more likely to use a health app than those who identified as white. Individuals earning between US $25,000 and US $74,999 per year were equally likely to use health apps. Likelihood of use increased by about 30% for those earning more than US $75,000 per year, but decreased for those earning less than US $25,000. There was a trend for increased use of health apps and higher BMI, with those who were obese being about 11% more likely to use apps than persons in the normal range. Cost was a significant concern among respondents, with a large proportion indicating that they would not pay anything for a health app. Interestingly, among those who had downloaded health apps, trust in their accuracy and data safety was quite high, and most felt that the apps had improved their health. About half of the app users (427/934, 45.7%) had stopped using some health apps, primarily due to high data entry burden, loss of interest, and hidden costs.

### Comparison With Prior Work

Our study provides a novel and meaningful contribution to the literature, as few prior studies have specifically examined the use of mobile health apps. While not as detailed as this survey, a 2012 Pew Research Center survey (n=3014) of mobile phone use provides some data to compare trends. The Pew survey found that 19% of mobile phone users had at least one health app, whereas the rate we found was significantly higher at 58.23% (934/1604) [10]. Similar to our findings, the Pew survey indicated that younger persons and those with higher incomes and education were more likely to use a health app. In contrast to our findings, the Pew survey found that women were more likely to use health apps. We also assessed additional variables not examined in the Pew survey, finding that health app use was higher among those identifying as Latino/Hispanic, and among those who were obese. As the prevalence of health app use has increased significantly from 19% to 58% since the Pew survey was conducted, the difference in use among men and women may have leveled out as more men adopted their use.

In terms of the most common reasons for health app use, our findings mirror those of two previous surveys indicating that exercise, nutrition, weight management, and blood pressure apps are most popular among consumers [7,10]. Interestingly, only 18% of respondents to a survey conducted by HealthMine stated they liked to learn health and wellness information from an app [7]. While we did not ask an exactly similar question, our data indicate a much higher regard for health apps, with respondents noting they found that apps improved their health and wanted increased features. Qualitative responses from our study indicated that consumers want improved health information capacity from their apps, with more specific and tailored health suggestions. This discrepancy may indicate that consumers have problems with the interfaces available on current apps rather than the concept of getting health information from apps in general.

### Strengths and Limitations

This study has number of strengths. First, the survey was conducted with a highly diverse national sample. In contrast to methods used by many online surveys, the quota sampling technique allowed us to collect data from groups that are typically under-represented in surveys of technology use: specifically, oversampling persons from racial/ethnic minority backgrounds, those with high school or less education, and those earning less than US $50,000 per year. Second, the survey assessed novel information, especially with regard to reasons for use and discontinuance of health apps, and analyzed demographic and health-related correlates of health app use. Third, the survey adhered to advanced online survey methodology, such as IP address verification, and employed a large sample size resulting in highly reliable estimates of survey responses (95% CI ± 2.5). In terms of limitations, the responses relied on self-report and included only persons who participate in panels managed by the survey company. In addition, these are cross-sectional data, and while helpful for examining health app usage at one point in time, it is likely that people vary their use patterns over time. These limitations should have minimal impact on the validity of the data, however, given the sampling and survey management techniques employed.

### Future Directions

Survey data from this study provides critical insight with regard to directions for the development of health-related mobile apps. While many people use health apps, a substantial portion of the population does not, primarily due to lack of interest or perceived need. Many consumers find that the abilities of apps remain limited. It appears that people are using apps to manage health conditions related to weight, which may be attributable to the proliferation at present of apps related to activity and nutrition, as well as use of mobile phone-connected wearable devices, such as the Fitbit and Apple Watch. The primary reason respondents stopped using apps was the demanding nature of data entry. Burdensome manual recording requirements are especially prominent in calorie- and nutrition-tracking apps. Open-ended responses indicated a strong interest in apps that would simplify their use and improve the ability of apps to track data without manual entry. Mobile monitoring devices are only in their infancy, primarily consisting of watches and similar bands (eg, Jawbone). Further development of more advanced and integrated products, such as biometric smart clothing [11] and even noninvasive sensor devices that continuously monitor end points such as blood glucose [12], will further the advancement of health apps by overcoming data entry burden. It also appears that many respondents were unaware of apps that accomplish some of the functions they wanted (eg, scanning a barcode to show nutritional information). This finding suggests that apps are generally difficult to find among the large number available, and supports the need for refereed clearinghouses that could help consumers evaluate features and make sense of available apps. Cost also appears to be a significant concern both for nonusers and users, with most people unwilling to pay anything for apps and discontinuing use when they find that in-app payments are required. Consumer aversion to cost may be due to the perception that mobile phone use and apps are primarily associated with communication and entertainment. While consumers are relying on their apps for health advice, they do not yet appear to see them as providing information that warrants payment.

App developers need to do more to promote the value and worth of their apps if they are to sustain a viable business model. This will likely necessitate increased openness to partnering with researchers to conduct well-designed trials examining the efficacy of health-directed apps. The state of the evidence for health apps is significantly lacking [4,13-15], limiting enthusiasm and perceived value among both consumers and health care professionals. Recommendations by health care providers could be influential in promoting health app adoption, but our results indicate that few health care providers currently advise health app use. This may be attributable to limited familiarity, but also to a lack of clinical trial evidence [16], which would likely be a foundational requirement for providers to feel comfortable that they are making sound recommendations to their patients [17]. Companies have attempted to create app formulary systems whereby providers can "prescribe" an app [18], but a disconnect remains between developers’ goals and the level of data that health care providers need in order to feel confident in recommending an app [19]. While not as rigorous, other techniques to promote value could involve review and ratings by expert panels such as those conducted by the UK’s National Health Service, which has launched a refereed library of health apps [20]. Additionally, academic/business partnerships such as those developed between Walgreens and Stanford Persuasive Technology Lab [21] could improve apps through increased integration of evidence-based principles into app design. Nevertheless, to truly be accepted within the health care infrastructure, apps will likely require clinical trial evidence in line with standard academic medical practices.

Our findings also suggest that a largely untapped market exists for apps that improve interactions with the health care system. Open-ended responses indicated a significant desire for apps that can help consumers coordinate their health care services. Consumers would like apps that can accomplish basic tasks such as making and being reminded of appointments and viewing information about their medications, as well as more complex capabilities such as syncing with their electronic health record and automatically uploading daily health status data. Consumers find that these features would allow them and their health care team to more accurately communicate and monitor their health. Integration, however, is important as consumers reported they do not want to toggle between multiple apps to accomplish these goals. Interest in syncing capabilities is also common among health care providers, 93% of whom noted that they would find value in apps connected to an EHR [22]. Health care integration capabilities of apps are likely lagging behind development of fitness and nutrition-focused apps due to data security and regulatory concerns among health care information technology professionals [23]. We also found high rates of distrust of data security among consumers who have not used a health app and dislike of apps that shared data with friend networks. Further penetration of apps into the health care sector will clearly require resolving information security and privacy issues, which is an objective of the Precision Medicine Initiative. Privacy is a significant issue for marketing and sponsorship of health apps with other recent surveys finding that few people would use an app sponsored by their employer insurance program, likely due to privacy concerns [7]. Thus, it appears that consumers would prefer health apps developed by private companies or trusted health care sources, such as hospitals or health care systems that operate separately from insurers. Results indicate that future work by researchers and app developers should include a focus on qualitative methods and usability testing to further define the requirements of quality apps and to ensure health apps meet expectations of end users.

### Conclusions

This research suggests that critical problems remain for the future of health apps. At present, apps are concentrated in the activity and weight-loss domain, which may limit perceptions of their utility for large portions of the population. Pricing and data entry problems also emerged as important concerns. App development by for-profit companies is a primary pathway for creating innovative products, but companies need to better respond to these user barriers in order for these products to reach a broader population. For health care systems, significant interest exists among users for communicating with doctors and using apps to seek health care-related services. The potential in this use of apps is great, and health care systems must embrace this technology and work through privacy and regulatory barriers to supply the services that patients are already requesting.

## Appendix

Multimedia Appendix 1 Sociodemographic and health characteristics of the sample.

Multimedia Appendix 2 Characteristics of health app use.

## Abbreviations

BMI: body mass index
EHR: electronic health record
GED: General Educational Development
RR: relative risk
VA: Veterans Affairs
