# Supplementary material for: Health App Use Among US Mobile Phone Owners: A National Survey
Source: JMIR Mhealth Uhealth. 2015 Nov 4;3(4):e101. doi: 10.2196/mhealth.4924 (PMC4704953; doi:10.2196/mhealth.4924)
Supplement: Multimedia Appendix 1 [file mhealth_v3i4e101_app1.pdf]

Table 1: Sociodemographic and health characteristics of the sample (n=1604)

**Item**	**Characteristic**	**N**	**%**
Sex	Female	795	49.56
	Male	795	49.56
	Transgender	14	0.88
Race/Ethnicity	African American or Black	408	25.44
	Asian American or Asian	114	7.11
	White or Caucasian	569	35.47
	Native American/Pacific islander	20	1.25
	Latino / Hispanic	447	27.87
	Other	46	2.87
Born in U.S.	No	158	9.85
	Yes	1445	90.09
	Don't know/Not sure	1	0.06
Employment	Working full-time	719	44.83
	Working part-time	212	13.22
	Not working	363	22.63
	Retired	213	13.28
	In school	97	6.05
Education	Less than 12th grade	79	4.93
	High school degree or GED	722	45.01
	Some college/vocational school/apprenticeship	399	24.88
	Bachelor’s degree	276	17.21
	Graduate degree (masters, PhD, MD, etc)	128	7.98
Household income	Less than $25,000	463	28.87
	$25,000 to $49,999	497	30.99
	$50,000 to $74,999	218	13.59
	$75,000 to $99,999	195	12.16
	$100,000+	231	14.40
Region of Country (n=1594)	Northeast	322	20.20
	Midwest	242	15.18
	South	636	39.90
	West	394	24.72
In general, would you say your health is:	Poor	44	2.74
	Fair	204	12.72
	Average	536	33.42
	Very good	603	37.59
	Excellent	217	13.53
Body Mass Index	<18.5 underweight	63	3.93
	18.5-24.9 normal	546	34.04
	25-29.9 overweight	438	27.31
	>=30 obese	557	34.73
Do you consider yourself to be…?	About the right weight	683	42.58
	Underweight	108	6.73
	Overweight	813	50.69
In a typical week, on how many days do you exercise or participate in physical activities for at least 15 minutes?	Never	263	16.40

	1 day	168	10.47
	2 days	348	21.70
	3-4 days	563	35.10
	5-7 days	262	16.33
In general, how healthy is your overall diet?	Poor	110	6.86
	Fair	320	19.95
	Good	596	37.16
	Very good	403	25.12
	Excellent	175	10.91
Do you smoke cigarettes?	Not at all	1056	65.84
	Some days	195	12.16
	Every day	353	22.01
Have you ever been diagnosed with any of the following conditions by a health professional?	No current medical diagnoses	807	50.31
	Hypertension (also called high blood pressure)	364	22.69
	High blood cholesterol	319	19.89
	Depression	267	16.65
	Obesity	198	12.34
	Diabetes (high blood sugar levels)	163	10.16
	Another chronic disease (e.g. auto-immune, etc.)	84	5.24
	Cancer	64	3.99
	COPD or Emphysema	62	3.87
	Heart Attack	51	3.18
	Stroke	51	3.18
	Substance use problem (alcohol or drugs)	45	2.81
	Ulcers	38	2.37
	Liver Disease	17	1.06
	HIV/AIDS	10	0.62
