# Supplementary material for: Health App Use Among US Mobile Phone Owners: A National Survey
Source: JMIR Mhealth Uhealth. 2015 Nov 4;3(4):e101. doi: 10.2196/mhealth.4924 (PMC4704953; doi:10.2196/mhealth.4924)
Supplement: Multimedia Appendix 2 [file mhealth_v3i4e101_app2.pdf]

Table 2. Characteristics of health app use

Item	Response Category	N	%
1.What brand of phone do you have? (n=1604)	iPhone	565	35.22
	Samsung	567	35.35
	HTC	96	5.99
	Nokia	63	3.93
	Blackberry	29	1.81
	Other	284	17.71
2. What company provides you cell phone service? (n=1604)	AT&T	420	26.18
	Verizon	342	21.32
	T-Mobile	311	19.39
	Sprint	177	11.03
	Boost	73	4.55
	Metro PCS	89	5.55
	Other:	192	11.97
3. Have you ever downloaded an "app" to track anything related to your health? (n=1604)	No	670	41.77
	Yes	934	58.23
4. How many health-related smartphone apps have you used? (n=934)	1-5 apps	545	58.4
	6-10 apps	104	11.1
	11-15 apps	67	7.2
	16- 20 apps	93	9.9
	21-25 apps	85	9.1
	26-30 apps	22	2.4
	30+ apps	18	1.9
5. Please check off all the reasons you have used health apps. (n=934)	Track how much activity/exercise I get	493	52.8
	Help me watch what I eat/improve what I eat	445	47.6
	Weight loss	437	46.8
	Show/teach me exercises	318	34.1
	Track a health measure (such as blood pressure, blood sugar)	266	28.5
	Track how much sleep I get	263	28.2
	Check my medical records/labs	216	23.1
	Help me relax (like a meditation or Yoga app)	210	22.5
	Keep a diary or log of my symptoms	189	20.2
	Help me stop a habit (such as smoking)	177	18.9
	Chat with my doctor/s or another health	173	18.5
	Remind me to take my medication (such blood pressure, HIV/AIDS)	168	18.0
	Access health information on symptoms, treatments, diagnoses, etc.	165	17.7
	I want to kill time when bored	66	7.1

	Other Reason	28	3.0
6. Rank the most important reasons you have not downloaded a health app (for those who answered No to Question 3). (n=670)	I'm just not interested in health apps	181	27.0
	They cost too much to buy	156	23.3
	I don't trust letting apps collect my data	103	15.4
	They would use too much of my data plan	85	10.9
	My health is fine and I don't need one	73	12.7
	They are too complicated/too much of a hassle to use	72	10.7
7. What would be the maximum amount you would pay for a health-related app? (n=1604)	I wouldn't pay anything	662	41.27
	Less than $1	108	6.73
	$1- $1.99	217	13.53
	$2-$3.99	183	11.41
	$4-$5.99	182	11.35
	$6-$9.99	139	8.67
	$10-$19.99	58	3.62
	$20+	55	3.43
8. On average, how often do you open or log on to use the health app you use most often? (n=934)	Less than once a month	59	6.3
	A few times a month	68	7.3
	A few times each week	195	20.9
	About 1 time each day	299	32.0
	2 or more times a day	313	33.5
9. On average, how many minutes do you spend using health-related smartphone apps on days that you use them? (n=934)	1-10 minutes	415	44.43
	11-30 minutes	415	44.43
	more than 30 minutes	104	11.13
10. How much do you trust that your health apps automatically record your data accurately? (n=934)	I don't use a health app that records my health data	23	2.5
	Don't trust them at all	11	1.2
	Somewhat distrust	52	5.6
	Neither trust nor distrust	88	9.4
	Moderately trust	416	44.5
	Very much trust	344	36.8
11. How much do you trust your health apps to keep your data safe and secure? (n=934)	Don't trust them at all	14	1.5
	Somewhat distrust	42	4.5
	Neither trust nor distrust	148	15.8
	Moderately trust	411	44
	Very much trust	319	34.2
12. How did you typically learn about the health apps you use? (n=934)	Searching the app store	327	35.0

	Friends or family	287	30.7
	Web searches (e.g. Google)	170	18.2
	TV	48	5.1
	Doctor, nurse or another health professional	38	4.2
	Newspaper/magazine	16	1.7
	From other apps	48	5.1
13. To what extent do you think health apps have improved your health? (n=934)	Made my health worse	25	2.7
	Didn't help at all	73	7.8
	Just a little bit improved	224	24.0
	Somewhat improved	339	36.3
	Very much improved	273	29.2
14. Which health apps do you currently have on your phone? (n=934)	Walgreens	123	13.2
	Fitbit	107	11.5
	Weight Watchers	59	6.3
	Web MD	36	3.9
	Nike+	34	3.6
	MyFitnessPal	33	3.5
	CVS	32	3.4
	MapMyFitnesss	31	3.3
	Loseit	25	2.7
	Noom	24	2.6
	Rite Aid	20	2.1
	Calorie Counter	20	2.1
	Pedometer/Steps	16	1.7
	Fooducate	15	1.6
	Runtastic	14	1.5
	Moves	14	1.5
	Blood Pressure	11	1.2
	MotionX247	7	0.7
	MapMyWalk	7	0.7
	Period Tracker	6	0.6
15. Are there any health apps you downloaded and no longer use? (n=427)	No	507	54.3
	Yes	427	45.7
16. What reasons do you no longer use them? (check all that apply) (n=427)	Takes too much time to enter data	190	44.5
	Lost interest	173	40.5
	There were hidden costs	154	36.1
	Too confusing to use	140	32.8
	I felt too connected to my friends/family/colleagues who used it too	124	29.0
	Didn’t help me as I wanted	81	19.0
	Found better apps	66	15.5
	I no longer need it/ I met my goals	44	10.3
	No longer works on my phone	44	10.3
	Other	11	2.6

17. How interested would you be in using an app to make appointments with your doctors? (n=1604)	I already use this	123	7.67
	Not very interested at all	350	21.82
	Just a little interested	211	13.15
	Somewhat interested	363	22.63
	Very interested	557	34.73
18. How interested would you be in using an app to look at your medical records? (n=1604)	I already use this	148	9.23
	Not very interested at all	290	18.08
	Just a little interested	169	10.54
	Somewhat interested	354	22.07
	Very interested	643	40.09
19. How interested would you be in using an app to write to your doctors? (n=1604)	I already use this	124	7.73
	Not very interested at all	353	22.01
	Just a little interested	196	12.22
	Somewhat interested	354	22.07
	Very interested	577	35.97
20. Has a doctor ever recommended you use a health app? (n=1031)	No	821	79.63
	Yes	210	20.37
